# The host protein Staufen1 interacts with the Pr55^Gag ^zinc fingers and regulates HIV-1 assembly via its N-terminus

**DOI:** 10.1186/1742-4690-5-41

**Published:** 2008-05-22

**Authors:** Laurent Chatel-Chaix, Karine Boulay, Andrew J Mouland, Luc DesGroseillers

**Affiliations:** 1 Département de biochimie, Université de Montréal, Montréal, Qc, Canada; 2 HIV-1 RNA Trafficking Laboratory, Lady Davis Institute for Medical Research-Sir Mortimer B. Davis Jewish General Hospital, Montréal, Qc, Canada; 3 Department of Medicine, McGill University, Montréal, Qc, Canada; 4 Department of Microbiology & Immunology, McGill University, Montréal, Qc, Canada

## Abstract

**Background:**

The formation of new infectious human immunodeficiency type 1 virus (HIV-1) mainly relies on the homo-multimerization of the viral structural polyprotein Pr55^Gag ^and on the recruitment of host factors. We have previously shown that the double-stranded RNA-binding protein Staufen 1 (Stau1), likely through an interaction between its third double-stranded RNA-binding domain (dsRBD3) and the nucleocapsid (NC) domain of Pr55^Gag^, participates in HIV-1 assembly by influencing Pr55^Gag ^multimerization.

**Results:**

We now report the fine mapping of Stau1/Pr55^Gag ^association using co-immunoprecipitation and live cell bioluminescence resonance energy transfer (BRET) assays. On the one hand, our results show that the Stau1-Pr55^Gag ^interaction requires the integrity of at least one of the two zinc fingers in the NC domain of Pr55^Gag ^but not that of the NC N-terminal basic region. Disruption of both zinc fingers dramatically impeded Pr55^Gag ^multimerization and virus particle release. In parallel, we tested several Stau1 deletion mutants for their capacity to influence Pr55^Gag ^multimerization using the Pr55^Gag^/Pr55^Gag ^BRET assay in live cells. Our results revealed that a molecular determinant of 12 amino acids at the N-terminal end of Stau1 is necessary to increase Pr55^Gag ^multimerization and particle release. However, this region is not required for Stau1 interaction with the viral polyprotein Pr55^Gag^.

**Conclusion:**

These data highlight that Stau1 is a modular protein and that Stau1 influences Pr55^Gag ^multimerization via 1) an interaction between its dsRBD3 and Pr55^Gag ^zinc fingers and 2) a regulatory domain within the N-terminus that could recruit host machineries that are critical for the completion of new HIV-1 capsids.

## Background

Human immunodeficiency type 1 (HIV-1) assembly consists in the formation of new viral particles which is the result of the radial multimerization of approximately 1,400 to 5,000 copies of the viral polyprotein Pr55^Gag ^(also named Gag) according to their quantification in mature or immature particles, respectively [1-3]. Pr55^Gag ^is thought to contain most of the determinants required for viral assembly since the expression of Pr55^Gag ^alone leads to the formation and release of virus-like particles (VLPs), structurally not really distinguishable from immature HIV-1 [4-6]. Pr55^Gag ^is a modular protein that contains 6 domains: matrix (MA), capsid (CA), nucleocapsid (NC), p6 and two spacer peptides, p2 and p1. Each of these domains plays specific roles during HIV-1 life cycle. During assembly, the MA domain, through its myristylated moiety and its highly basic domain, anchors assembly complexes to membranes [4-6]. Whether assembly takes place at the inner leaflet of the plasma membrane or at the multivesicular bodies (or both) is still under debate [7-17].

Pr55^Gag ^multimerization is likely initiated by NC/NC contacts [18,19] probably when Pr55^Gag ^is still in a cytosolic compartment [20-23]. The basic amino acid stretch present in NC is thought to non-specifically recruit RNA that serves as a scaffold for multimerizing Pr55^Gag ^[24-26]. Indeed, mutations abrogating the global positive charge of this sub-domain compromise viral assembly [24,25]. NC also possesses two zinc fingers that are important for the specific packaging of HIV-1 genomic RNA [27-29]. Recently, Grigorov *et al. *demonstrated the involvement of both NC zinc fingers in Pr55^Gag ^cellular localization and HIV-1 assembly [30]. Similarly, the first NC zinc finger was shown to be part of the minimal Pr55^Gag ^sequence required for multimerization (called the I domain) [5,6]. Since NC function during assembly can be mimicked by its substitution with a heterologous oligomerization domain [31,32], NC/NC contacts probably serve as a signal for the higher order multimerization of Pr55^Gag ^under the control of other domains. Indeed, the C-terminal third of the CA domain and the spacer peptide p2 are part of the I domain and have been shown by mutagenesis and structural analyses to be also very important players during HIV-1 assembly [26,33-42].

The HIV-1 assembly process within the cell appears to be tightly regulated in time and space and relies on the sequential acquisition and release of host proteins that are required for the cellular localization, multimerization and budding of new capsids [4,43]. For instance, the ATP-binding protein ABCE1/HP68 is important for the completion of Pr55^Gag ^multimerization via a transient interaction with the NC domain of Pr55^Gag ^[44-47]. Adaptor proteins 1, 2, 3 (AP-1; AP-2; AP-3) are involved in Pr55^Gag ^intracellular trafficking through their association with the MA domain of Pr55^Gag ^[12,48,49]. Finally, endosomal sorting complex required for transport (ESCRT)-I and -III machineries are recruited by the p6 domain of Pr55^Gag ^and are crucial for the budding and release of the neosynthesized viral particles [50].

Staufen1 (Stau1) is also a Pr55^Gag^-binding protein that influences HIV-1 assembly [51-53]. Stau1 belongs to the double-stranded RNA-binding protein family [54,55] and is involved in various cellular processes related to RNA. Stau1 was first studied for its role in the transport and localization of mRNAs in dendrites of neurons [56]. More recently, Stau1 was identified as a central component of a new mRNA decay mechanism termed Staufen-mediated decay [57]. In addition to its functions in RNA localization and decay, Stau1 can also stimulate translation of repressed messengers containing structured RNA elements in their 5'UTR [58].

Stau1 is a host factor that is selectively encapsidated into HIV-1 [53]. Stau1 co-purifies with HIV-1 genomic RNA and interacts with the NC domain of Pr55^Gag ^[52,53] suggesting that Stau1 assists NC's functions during the HIV-1 replication cycle. Stau1 levels in the producer cells are important for HIV-1 since both Stau1 overexpression and depletion using RNA interference affect HIV-1 infectivity [52,53]. In addition to a putative role in HIV-1 genomic RNA packaging [53], we recently showed that Stau1 modulates HIV-1 assembly by influencing Pr55^Gag ^multimerization [51]. Indeed, using a new Pr55^Gag ^multimerization assay relying on bioluminescence resonance energy transfer (BRET), we demonstrated that both Stau1 overexpression and depletion enhanced multimerization and consequently increased VLP production. Although Stau1 and Pr55^Gag ^interact in both cytosolic and membrane compartments, this effect of Stau1 on Pr55^Gag ^oligomerization was only observed in membranes, a cellular compartment in which Pr55^Gag ^assembly primarily occurs. However, the mechanism by which Stau1 influences HIV-1 assembly at the molecular level remains unknown although it is likely that it relies on the Stau1 interaction with HIV-1 Pr55^Gag^.

Using co-immunoprecipitation and BRET assays, we showed that both Pr55^Gag ^NC zinc fingers are involved in Stau1/Pr55^Gag ^interaction as does the Stau1 dsRBD3 [52]. Unexpectedly, we found that the binding of Stau1 to NC is not sufficient per se to fully enhance Pr55^Gag ^multimerization. To determine which domain of Stau1 modulates the HIV-1 Pr55^Gag ^multimerization process, we analyzed several Stau1 deletion mutants for their capacity to enhance Pr55^Gag ^multimerization. Using the Pr55^Gag^/Pr55^Gag ^BRET assay either in live cells or after cell fractionation, we showed that the first 88 amino acids at the N-terminal of Stau1 confer the capacity to enhance both Pr55^Gag ^multimerization and VLP production. Although unable to enhance multimerization, this mutant was still able to interact with Pr55^Gag^. This study provides important new information about the molecular determinants required for Stau1 function in HIV-1 assembly.

## Methods

### Cell culture and reagents

Human embryonic kidney fibroblasts (HEK 293T) were cultured in Dulbecco's Modified Eagle Medium (Invitrogen) supplemented with 10% cosmic calf serum (HyClone) and 1% penicillin/streptomycin antibiotics (Multicell). Transfections were carried out using either the calcium phosphate precipitation method or the Lipofectamine 2000 reagent (Invitrogen). For Western blots, mouse and rabbit HRP-coupled secondary antibodies were purchased from Dako Cytomation and signals were detected using the Western Lightning Chemiluminescence Reagent Plus (PerkinElmer Life Sciences). Signals were detected with a Fluor-S MultiImager apparatus (Bio-Rad). Anti-Na-K ATPase antibodies were kindly provided by Dr. Michel Bouvier.

### Plasmid construction

The construction of pcDNA3-RSV-Stau1^55^-HA_3_, pcDNA3-RSV-Stau1^F135A^-HA_3_, pcDNA-RSV-Stau1^ΔNt88^-HA_3_, pCMV-Stau1^55^-YFP, pCMV-Stau1^F135A^-YFP, pCMV-Stau1^ΔNt88^-YFP, pCMV-Stau1^ΔdsRBD3^-YFP, pCMV-Pr55^Gag^-*R*luc, pCMV-Pr55^Gag^-YFP, pCMV-NC-p1-YFP and pCMV-CA-p2-NC-p1-*R*luc was reported before [51-54,59]. The HxBRU PR-provirus and the Rev-independent Pr55^Gag ^expressor were described before [51,53,60].

To construct pcDNA-RSV-Stau1^ΔNt37^-HA_3_, a polymerase chain reaction (PCR) was performed using pcDNA3-RSV-Stau1^55^-HA_3 _as template, sense (5'-ATCAGGTACCATGGGTCCATTTCCAGTTCCACCTTT-3') and anti-sense (5'-CACATCTAGATCATTTATTCAGCGGCCGCACTGAGCAGCGT-3') oligonucleotide primers and the Phusion DNA polymerase (New England Biolabs). The PCR product was purified and digested with *Kpn*I and *Xba*I restriction enzymes (Fermentas) and then cloned into the *Kpn*I/*Xba*I cassette of pCDNA3-RSV.

To generate pcDNA-RSV-Stau1^55^-Flag plasmid, oligonucleotides (5'-GGCCTTGATTACAAGGATGACGATGACAAG-3' and 5'-GGCCCTTGTCATCGTCATCCTTGTAATCAA-3') were hybridized and then inserted into the *Not*I sites of pcDNA-RSV-Stau1^55^-HA_3 _in replacement of the HA-tag. For the construction of pcDNA-RSV-Stau1^ΔNt88^-Flag, the *EcoR*I fragment of pcDNA-RSV-Stau1^ΔNt88^-HA_3 _that contained the mutated Stau1 sequence, was cloned into *EcoR*I-digested pcDNA-RSV-Stau1^55^-Flag plasmid.

The expressors of NC-p1-YFP and Pr55^Gag^-YFP mutants were PCR amplified using the PCR all-around technique [59] to generate the following mutations: the C15S mutation was introduced with the primer pair 5'-AAGAGTTTCAATTGTGGCAAA-3' and 5'-GAAACTCTTAACAATCTTTCT-3'; the C49S mutation was generated with the primer pair 5'-GATAGTACTGAGAGACAGGCT-3' and 5'-AGTACTATCTTTCATTTGGTG-3'; R7S, R10S and K11S mutations (R7 mutant) were introduced with the primer pair 5'-TTTAGCAACCAAAGCTCGATTGTTAAGTGTTTC-3' and 5'-AATCGAGCTTTGGTTGCTAAAATTGCCTCTCTG-3'. PCR reactions were carried out with the Phusion enzyme (New England Biolabs) at 95°C for 50 s, 55°C for 60 s and 72°C for 90 s, for 18 cycles. Resulting products were incubated with 10 units of *Dpn*I enzyme (Fermentas) and then transformed into competent bacteria. Positive clones containing the mutation(s) were screened by restriction and sequencing analyses. The double zinc fingers mutant expressors (pCMV-Pr55^Gag C15–49S^-YFP and pCMV-NC-p1^C15–49S^-YFP) were generated by PCR with the oligonucleotide primer pair for the C49S mutation using the corresponding plasmids that contain the C15S mutation.

### Membrane flotation assays and S100-P100 fractionation

Forty hours post-transfection, cell extracts were prepared by passing the cells 20 times through a 23G1 syringe in TE (10 mM Tris pH7.4, 1 mM EDTA pH 8) containing 10% sucrose and proteases inhibitors (Roche). Nuclei were removed by centrifugation at 1,000 × g. Resulting cytoplasmic extracts were separated using the membrane flotation assay as previously described [51]. Membrane-associated complexes were collected (fractions 2 and 3). Membranes were solubilized by treating these complexes with 0.5% Triton X-100 at room temperature for 5 minutes and samples were subjected to S100/P100 fractionation as previously described [51] by ultracentrifugation at 100,000 × g for 1 h at 4°C. Supernatants (S100 fractions) and pellets (P100 fractions) were collected and analyzed by Western blotting using anti-CA, anti-HA and anti-Na-K ATPase mouse antibodies.

### BRET assays

293T cells were transfected in 6-well plates with constant amounts of the *R*luc-fused energy donor expressor (25–75 ng), increasing amounts of YFP-fused acceptor expressor (0.25–2 μg) and Stau1-HA_3_-expressing plasmid (1–1.5 μg) when indicated. 48 hours post-transfection, cells were collected in PBS-EDTA 5 mM and diluted to approximately 2 × 10^6 ^cells/mL. BRET assays were performed as described before [51,52] using a Fusion α-FP apparatus (Perkin-Elmer). In this interaction assay, an X-*R*luc fusion protein is used as an energy donour whereas a Y-YFP fusion protein is an energy acceptor. When the two fusion proteins are in close proximity (< 100Å), non-radiative resonance energy is transferred from X-*R*luc to Y-YFP which in turn emits measurable fluorescence. This can be quantified by the calculation of the BRET ratio which allows detection of protein-protein interactions. The BRET ratio was defined as [(emission at 510 to 590 nm)-(emission at 440 to 500 nm) × Cf]/(emission at 440 to 500 nm), where Cf corresponds to (emission at 510 to 590 nm)/(emission at 440 to 500 nm) when *R*luc fused protein is expressed alone. The total YFP activity/*R*luc activity ratio reflects the relative levels of the two fusion proteins in the cells. The BRET ratio increases with the total YFP activity/*R*luc activity ratio since more YFP-fused molecules bind to *R*luc-fused proteins. For Pr55^Gag ^multimerization assays, in order to avoid misinterpretation due to variations in relative levels of the Pr55^Gag ^fusion proteins, changes in the Pr55^Gag^/Pr55^Gag ^BRET ratios following Stau1 overexpression were always analyzed at similar total YFP activity/*R*luc activity ratio.

When Pr55^Gag^/Pr55^Gag ^BRET assays were performed following membrane flotation assays, the *R*luc substrate coelenterazine H (NanoLight Technology) was added to 90 μL of each fraction and BRET ratio was determined as in live cells. BRET ratios in fractions 1, 3, 4, 5 and 6 were not considered because luciferase activity was too low in these fractions and hence, did not lead to the determination of a reliable BRET ratio.

For CA-p2-NC-p1-*R*luc/Stau1-YFP and Stau1^55^-*R*luc/NC-p1-YFP interaction assays, BRET ratios were always compared at similar total YFP activity/*R*luc activity ratio. The BRET ratio determined in the context of the expression of the unfused YFP protein (YFP) corresponds to non specific interactions between the energy donor and the YFP. Hence, this background BRET ratio was always subtracted from all BRET ratios and was set to 0%. The BRET ratio determined following co-expression of the energy donor and the wild type energy acceptor was set to 100%.

For dose-response Pr55^Gag^/Pr55^Gag ^BRET assays, 293T cells were transfected with fixed amounts of pCMV-Pr55^Gag^-*R*luc and pCMV-Pr55^Gag^-YFP and increasing amounts (0.25–2 μg) of different Stau1-HA_3 _expressors. BRET assays were performed 48 hours post-transfection as described above.

### Co-immunoprecipitation assays

293T cells were transfected with Stau1^55^-flag and Gag expressors using Lipofectamine 2000 (Invitrogen). Twenty hours post-transfection, cells were collected in lysis buffer (150 mM NaCl, 50 mM Tris pH 7.4, 1 mM EDTA, 1% Triton X-100) containing proteases inhibitors (Roche). Each cell lysate (1.5 mg of proteins) was pre-cleared with IgG-agarose (Sigma-Aldrich) for 1 h at 4°C and then subjected to immunoprecipitation using 15 μL of anti-Flag M2 affinity gel (Sigma-Aldrich) for 2 h at 4°C. Immune complexes were washed 3 times during 5 minutes with cold lysis buffer, eluted with the Flag peptide (Sigma-Aldrich), resolved in SDS-containing acrylamide gels and analyzed for their content in Stau1 and Gag proteins by Western blotting using mouse monoclonal anti-Flag (Sigma-Aldrich), anti-GFP (Roche) and anti-CA antibodies.

### Virus-like particle purification

293T cells were transfected with Stau1^55^-HA_3 _and Gag expressors using Lipofectamine 2000 (Invitrogen). Twenty hours post-transfection, supernatants were collected and cleared through a 0.45 μm filter. VLPs were pelleted through a sucrose cushion (20% in Tris-NaCl buffer) by ultracentrifugation during 1 hour at 220,000 × g. VLPs were resuspended in Tris-NaCl buffer and analyzed by Western blotting using anti-CA antibodies. Pr55^Gag ^signals in the VLPs and the cell extracts were quantitated using the Quantity One (version 4.5) software (Bio-Rad).

## Results

### Both NC zinc fingers mediate Stau1/Pr55^Gag ^interaction

**Figure 1:**
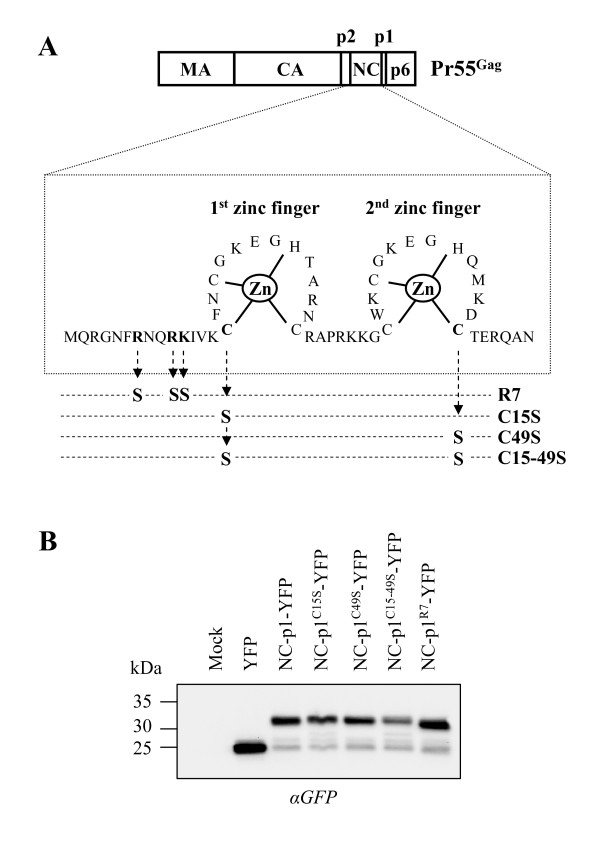
**Design and expression of NC mutants used for the fine mapping of Stau1/NC interaction**. **(A) **Schematic representation of Pr55^Gag ^with emphasis on the sequence of NC and its two zinc fingers. Several point mutations were introduced in the basic region or in the zinc fingers of NC-p1-YFP fusion protein to generate four mutants. **(B) **293T cells were transfected with YFP, NC-p1-YFP and mutated NC-p1-YFP expressors. 48 hours post-transfection, cell lysates were prepared and analyzed by Western blotting using anti (α)-GFP antibodies.

The interaction between Stau1 and Pr55^Gag ^is likely a critical determinant for Stau1 function in HIV-1 assembly. Indeed we previously showed that a single point mutation in the third double-stranded RNA-binding domain of Stau1 (Stau1^F135A^) prevented both the association of the mutant to Pr55^Gag ^and the Stau1-mediated increase of HIV-1 assembly [51-53]. Moreover, we showed that Stau1/Pr55^Gag ^interaction required the NC domain [52] that contains motifs involved in several steps during HIV-1 assembly. As a first step, to understand the molecular mechanisms underlying Stau1 influence on HIV-1 assembly, we identified which NC sub-domain is required for Pr55^Gag^/Stau1 association using the BRET assay with Stau1^55^-*R*luc and wild type or mutant NC-p1-YFP fusion proteins. Four NC mutants were constructed. Point mutations were introduced in the NC-p1-YFP fusion protein to disrupt the first zinc finger (NC-p1^C15S^-YFP), the second (NC-p1^C49S^-YFP), both zinc fingers (NCp1-YFP^C15–49S^) or the N-terminal basic residues (NCp1-YFP^R7^)(Figure 1A). For this mutant, Arg^7^, Arg^10 ^and Lys^11 ^were substituted for serines (Figure 1A). Mutations in this basic region were previously reported to severely affect HIV-1 assembly [24]. Constructs encoding the wild type and mutants NC fusion proteins were transfected in 293T cells and their expression patterns were analyzed by Western blotting using an anti-GFP antibody. Figure 1B shows that wild type and mutant NC-p1-YFP proteins were well expressed and have the expected molecular weight. However, for unknown reasons, NC-p1^C15–49S^-YFP was always slightly less expressed than the other NC-p1-YFP proteins.

**Figure 2:**
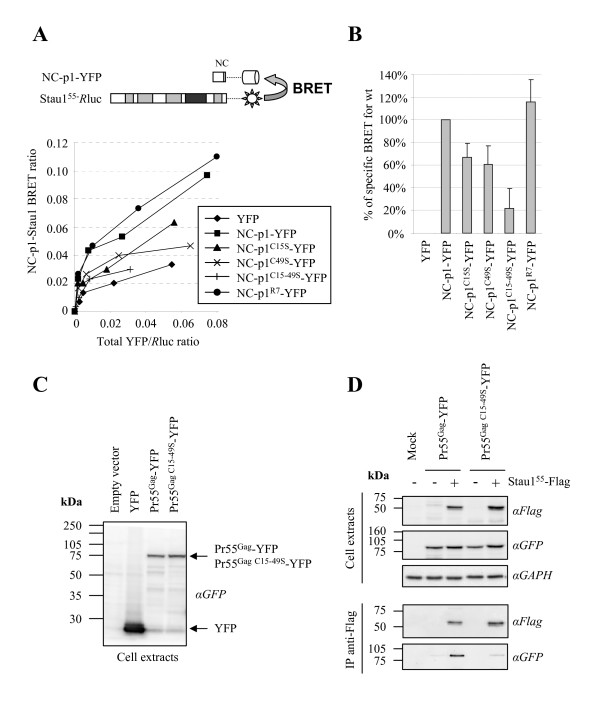
**The NC zinc fingers mediate Stau1/Pr55^Gag ^interaction**. **(A) **Top: schematic representation of the Stau1/NC-p1 BRET assay. Bottom: 293T cells were transfected with constant amounts of pCMV-Stau1^55^-*R*luc and increasing amounts of wild type or mutated NC-p1-YFP expressors. 48 hours post-transfection, BRET ratios were determined and plotted in function of their corresponding total YFP/*R*luc ratio which allows us to compare BRET ratios at the same relative expression levels of fusion proteins. This figure is representative of four independent experiments. **(B) **BRET ratios were compared at identical total YFP/*R*luc ratio and corrected by subtracting the background BRET ratio calculated for unfused YFP and Stau1^55^-*R*luc co-expression (see Methods). The corrected BRET ratio for Stau1^55^-*R*luc and wild type NC-p1-YFP coexpression was arbitrarily set to 100%. These results are representative of four independent experiments. **(C) **293T cells were transfected with Pr55^Gag^-YFP or Pr55^Gag C15-49S^-YFP expressors. Twenty hours post-transfection, lysates were analyzed by Western blotting using anti-GFP antibodies. **(D) **Following Stau1^55^-Flag and wild-type or mutated Pr55^Gag^-YFP co-expression, 293T cell lysates were submitted to immunoprecipitation using anti-Flag antibodies. Immune complexes were analyzed for their content of YFP-fused proteins and Stau1-Flag using anti (α)-GFP and anti (α)-Flag antibodies, respectively. Anti (α)-GAPDH antibodies were used as loading controls. This figure is representative of four independent experiments.

These proteins were then tested for their capacity to interact with Stau1^55 ^using the BRET assay in live 293T cells (Figure 2A). This technique allows us to detect protein-protein interaction in live cells between *R*luc-fused Stau1 and NC-p1-YFP molecules (Figure 2A). Indeed, when the two fusion proteins are in close proximity (≤ 100Å) as a consequence of Stau1-NC interaction, non-radiative resonance energy is transferred from the emitting *R*luc to YFP which becomes excited and in turn emits fluorescence. A BRET ratio is calculated for each condition (see Methods). To perform BRET saturation experiments, we transfected 293T cells with constant amounts of pCMV-Stau1^55^-*R*luc plasmid and increasing amount of different NC-p1-YFP expressors. BRET assays were performed 48 hours post-transfection (Figures 2A, B). BRET saturation experiments allowed us to compare BRET ratios at the same relative ratio between fusion proteins (comparable total YFP/*R*luc ratio) (Figure 2B). As expected, we readily detected a specific BRET between wild type NC-p1-YFP and Stau1^55^-*R*luc (arbitrarily set to 100% in Figure 2B) as compared to co-expression of Stau1^55^-*R*luc and YFP alone (Figures 2A, B). Mutations that modify the NC N-terminal basic region did not affect the binding of NC to Stau1 since the saturation profile for Stau1/NC-p1^R7^-YFP BRET was almost identical to the one obtained with Stau1/NC-p1-YFP (Figures 2A, B). In contrast, when the two zinc fingers were mutated (NC-p1^C15–49S^-YFP), the BRET saturation profile was comparable to that obtained with YFP alone and hence, mostly attributable to background (Figure 2A). When compared to NC-p1-YFP at the same total YFP/*R*luc ratio, the corrected BRET ratio was decreased by 80% (Figure 2B). This suggests that NC-p1^C15–49S^-YFP lost almost completely its ability to interact with Stau1. Mutations in individual zinc finger (NC-p1^C15S^-YFP and NC-p1^C49S^-YFP) only affected the BRET ratio by 30–40% and these two mutants showed an intermediate profile (Figures 2A, B).

This suggests that the integrity of at least one NC zinc fingers is required for Stau1/NC interaction.

We used a second technique to confirm the involvement of both zinc fingers in Stau1-NC interaction in the context of full-length Pr55^Gag^. We generated a Pr55^Gag^-YFP-expressing plasmid in which both zinc fingers were mutated (Pr55^Gag C15–49S^-YFP)(see below). As shown in Figure 2C, this mutant was expressed to the same level as the wild-type Pr55^Gag^-YFP and migrated in SDS-containing acrylamide gels at the expected molecular weight (80 kDa). Following co-expression of Flag-tagged Stau1^55 ^with wild type or mutated Pr55^Gag^-YFP in 293T cells (Figure 2D, upper panel), Stau1^55^-Flag-containing complexes were immunoprecipitated using anti-Flag antibodies. Immunopurified material was analyzed by Western blot using monoclonal anti-GFP and anti-Flag antibodies (Figure 2D, lower panel). As expected, Pr55^Gag^-YFP successfully co-precipitated with Stau1^55^-Flag. In contrast, despite similar levels of expression in the cell (Figure 2D, upper panel), the Pr55^Gag C15–49S^-YFP mutant was not efficiently co-immunoprecipitated with Stau1^55^-Flag as compared to wild type (Figure 2D, lower panel) suggesting that the association between this mutant and Stau1^55 ^is impaired. Pr55^Gag C15S^-YFP and Pr55^Gag C49S^-YFP mutants retained some association with Stau1^55^-Flag although they displayed lower binding capability than the wild type Pr55^Gag ^(not shown), consistent with the BRET assay. Altogether, these results show that the two zinc fingers within the NC domain of Pr55^Gag ^mediate its association with Stau1. Moreover, this suggests that Stau1 influences those assembly processes that depend on NC zinc fingers.

### Mutations in the NC zinc fingers severely compromises Pr55^Gag ^multimerization and release

**Figure 3:**
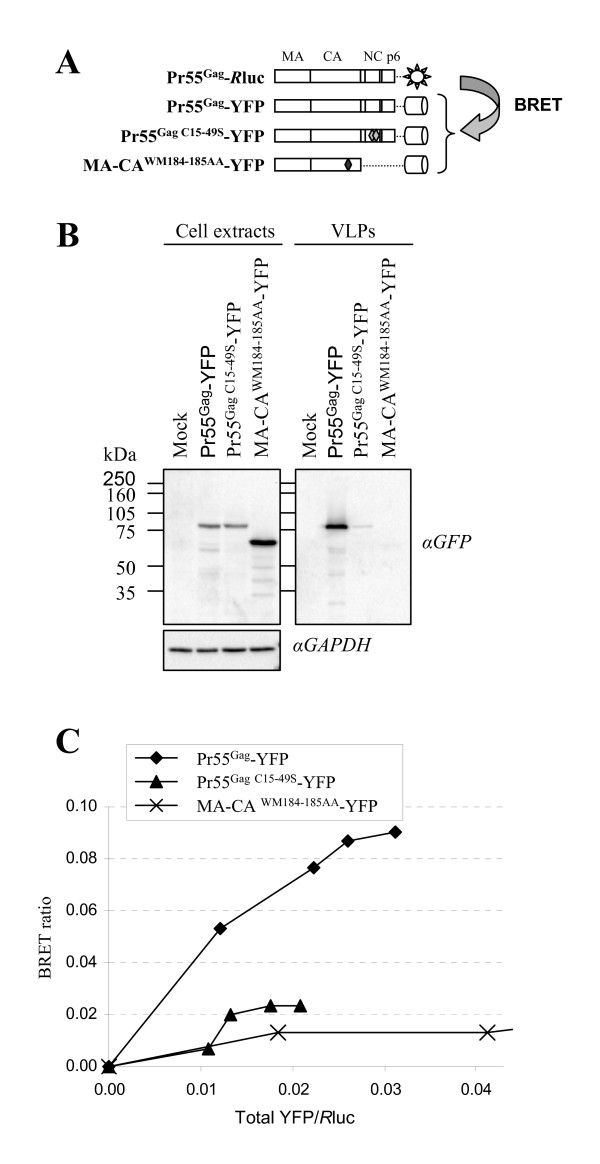
**Disruption of both Pr55^Gag ^zinc fingers affects VLP production**. **(A) **Schematic representation of the Gag fusion proteins used in the BRET and release assays. **(B) **Wild-type or mutated YFP-fused Gag proteins were expressed in 293T cells for twenty-four hours. VLPs in the cell supernatant were purified. Cell lysates and VLPs were analyzed by Western blotting using anti (α)-GFP antibodies. Anti (α)-GAPDH antibodies were used as loading controls. This figure is representative of three independent experiments. **(C) **293T cells were transfected with constant amounts of pCMV-Pr55^Gag^-*R*luc and increasing amounts of wild type or mutated YFP-fused Gag expressors. Twenty-four hours post-transfection, cells were collected and BRET ratios determined. BRET ratios are plotted in function of their corresponding total YFP/*R*luc ratio. This figure is representative of three independent experiments.

The fact that Stau1 influences HIV-1 Pr55^Gag ^multimerization and associates with NC zinc fingers is consistent with previous reports showing that these structural motifs are important in HIV-1 assembly [29,30,46]. To confirm this hypothesis in a system that tests direct interaction, we evaluated the consequence of mutations in the Pr55^Gag ^zinc fingers on VLP release and on Pr55^Gag ^dimerization using the BRET assay. 293T cells were transfected with Pr55^Gag^-YFP and Pr55^Gag C15–49S^-YFP expressors (Figure 3A). Twenty-four hours post-transfection, VLPs were collected from the supernatant and cells were collected. In the cell extracts, Pr55^Gag^-YFP and Pr55^Gag C15–49S^-YFP were present at similar levels (Figure 3B, left panel). In contrast, the release of Pr55^Gag C15–49S^-YFP in the cell supernatant was reduced by 95.1% (+/- 3.4 S.D.; n = 3) as compared to that of Pr55^Gag^-YFP (Figure 3B, right panel) suggesting that this mutant failed to efficiently assemble. Used as a negative control, MA-CA^WM184–185AA^-YFP (Figure 3A), a Pr55^Gag ^mutant that was shown to be almost completely monomeric in the cell and unable to generate VLPs [26,51], was not detected in the cell supernatant although it was expressed at higher levels than Pr55^Gag^-YFP and Pr55^Gag C15–49S^-YFP in the cell (Figure 3B).

Then, we determined whether mutations in the zinc fingers affect Pr55^Gag^multimerization. Using the BRET assay in live cells, we tested the capacity of Pr55^Gag C15–49S^-YFP to dimerize with Pr55^Gag^-*R*luc, the wild-type Pr55^Gag^-YFP being used as control (Figure 3A). As shown in Figure 3C, Pr55^Gag ^homo-dimerization was readily detectable with a BRET ratio of 0.09 at saturation. In contrast, Pr55^Gag C15–49S^-YFP failed to interact with Pr55^Gag^-*R*luc in the BRET assay since its saturation curve was similar to the one obtained with the monomeric Gag mutant MA-CA^WM184–185AA^-YFP. Altogether, these results clearly show that, in the context of VLP assembly, Pr55^Gag ^zinc fingers are important for multimerization and release. This suggests that Stau1, through its binding to the NC zinc fingers could influence crucial processes that are controlled by these motifs during HIV-1 assembly.

### The N-terminal domain of Stau1 is required for the Stau1-mediated enhancement of Pr55^Gag ^multimerization

**Figure 4:**
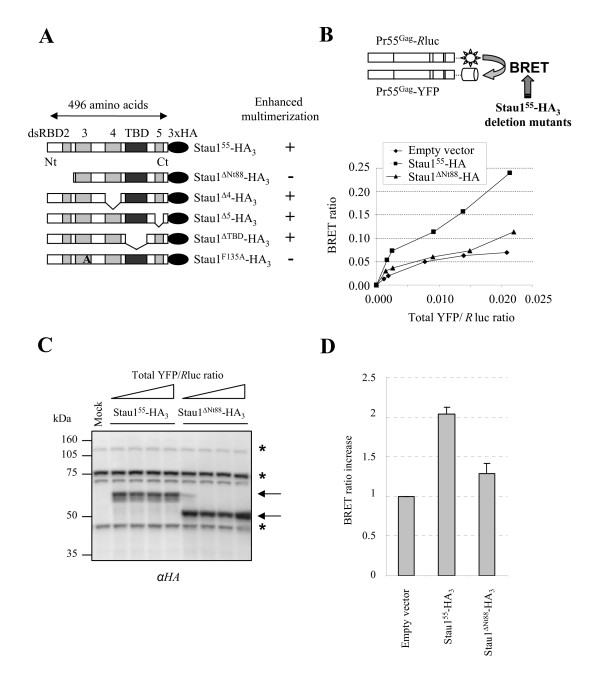
**The N-terminus of Stau1 is required for the modulation of Pr55^Gag ^multimerization in live cells**. **(A) **Schematic representation of HA-tagged Stau1^55 ^expressors. Stau1 double-stranded RNA-binding domains (dsRBD) and tubulin-binding domain (TBD) are represented as grey and black boxes, respectively. **(B) **Schematic representation of the Pr55^Gag^/Pr55^Gag ^BRET assay. This assay is used as a sensor of Pr55^Gag ^multimerization. 293T cells were transfected with constant amounts of pCMV-Pr55^Gag^-*R*luc and increasing amounts of pCMV-Pr55^Gag^-YFP. A constant amount of a third plasmid expressing Stau1^55^-HA_3 _or Stau1^ΔNt88^-HA_3 _was included in the transfection procedure. *R*luc activity as well as transmitted and total YFP activities was measured. BRET ratios were plotted in function of their corresponding total YFP/*R*luc ratio which allows us to compare BRET ratios at the same relative expression levels of Pr55^Gag ^fusion proteins. This figure is representative of four independent experiments. **(C) **Cells corresponding to the four last points of each curve from Figure 4B were lysed. Cell lysates were analyzed by Western blotting using anti (α)-HA antibodies for their content in over-expressed Stau1 proteins. *: Non-specific labelling typically obtained with the anti-HA antibody. **(D) **BRET ratios were compared at comparable total YFP/*R*luc ratio. The BRET ratio corresponding to the pr55^Gag ^fusions expressed alone was arbitrarily set to 1. The BRET induction levels were then determined and are shown in the graph. These results are representative of 4 experiments.

We previously showed that Stau1 over-expression or depletion from cells enhanced Pr55^Gag ^multimerization. To determine if the binding of Stau1 to NC is sufficient for Pr55^Gag ^multimerization or whether other determinants within Stau1 are required for this process, we tested several Stau1 deletion mutants for their capacity to enhance assembly (Figure 4A). To this end, we used the previously described Pr55^Gag^/Pr55^Gag ^BRET assay in live 293T cells as a sensor for changes in Pr55^Gag ^multimerization (Figure 4B)[51]. Indeed, *R*luc- and YFP-fused Pr55^Gag ^co-expression generates a positive BRET ratio in live cells as a consequence of Pr55^Gag ^multimerization. In order to compare BRET ratio changes at the same relative levels of Pr55^Gag ^fusion proteins, we performed BRET saturation experiments. As previously reported, when Stau1^55^-HA_3 _was co-expressed with Pr55^Gag^-*R*luc and Pr55^Gag^-YFP in 293T cells, the Pr55^Gag^/Pr55^Gag ^BRET ratio increased as a consequence of enhanced Pr55^Gag ^multimerization (Figures 4B)[51]. Several HA-tagged Stau1 deletion mutants were then tested for their capacity to enhance Pr55^Gag^/Pr55^Gag ^BRET ratio and hence, Pr55^Gag ^multimerization. Interestingly, Stau1^ΔNt88^-HA_3_, a mutant that lacks the dsRBD2 as a consequence of the deletion of the first N-terminal 88 amino acids (Figure 4A) was unable to significantly increase the Pr55^Gag^/Pr55^Gag ^BRET ratio in live cells [1.29 (+/-0.13 S.D. n = 4)-fold induction] as compared to Stau1^55^-HA_3 _[2.04 (+/-0.09 S.D. n = 4)-fold induction] (Figures 4B, D). Western blot analyses showed that Stau1^ΔNt88 ^was expressed at levels comparable to that of wild type Stau1-HA_3 _(Figure 4C). Nevertheless, a moderate increase in Pr55^Gag ^multimerization was seen when Stau1^ΔNt88 ^was highly over-expressed although its effect on Pr55^Gag ^multimerization was always weaker than that obtained with Stau1^55^-HA_3 _(see below). In contrast, mutants with deletion in dsRBD4, dsRBD5 or tubulin-binding domain (TBD) all enhanced the Pr55^Gag^/Pr55^Gag ^BRET ratio at levels comparable to that obtained with Stau1^55^-HA_3 _(data not shown). As control, Stau1^F135A^-HA_3_, a Stau1 mutant that does not bind Pr55^Gag^, failed to stimulate Pr55^Gag ^multimerization (data not shown)[51]. Therefore, the Stau1-mediated enhancement of Pr55^Gag ^multimerization requires two determinants: dsRBD3 for the association with NC and the N-terminus.

### Stau1^ΔNt88 ^still interacts with HIV-1 Gag

**Figure 5:**
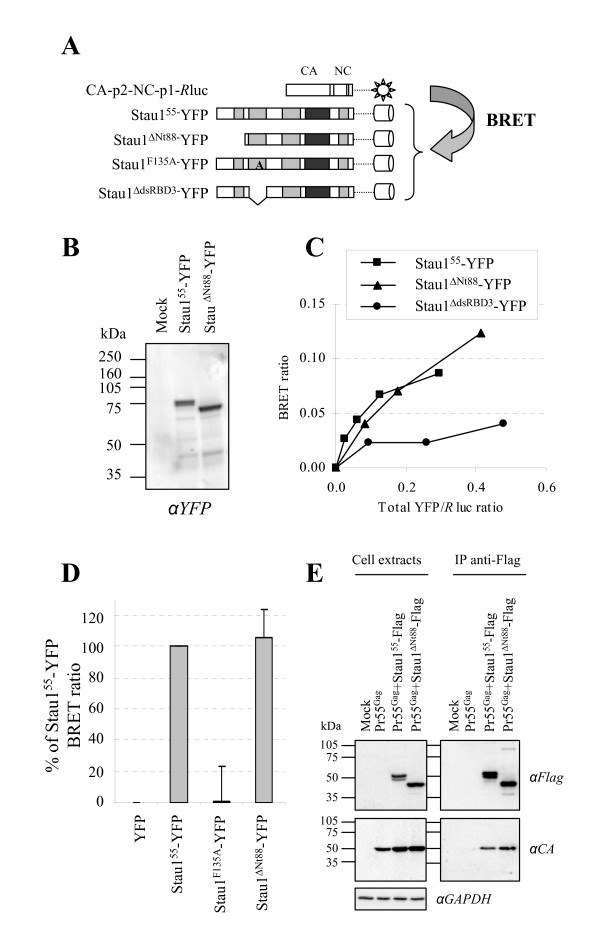
**Stau1^ΔNt88 ^interacts with HIV-1 Gag in live cells**. **(A) **Schematic representation of the Stau1/CA-p2-NC-p1 BRET assay. **(B) **293T cells were cotransfected with CMV-CA-p2-NC-p1-*R*luc and Stau1-YFP expressors. 48 hours post-transfection, Stau1^55^-YFP and Stau1^ΔNt88^-YFP contents in the cells were analyzed by Western blotting using anti (α)-GFP antibodies. **(C) **293T cells were transfected with constant amounts of CA-p2-NC-p1-Rluc-expressing plasmid and increasing amounts of wild-type or mutated YFP-fused Stau1 expressors. Twenty four hours post-transfection, live transfected cells were used for CA-p2-NC-p1/Stau1 BRET assays. BRET ratios are plotted in function of their corresponding total YFP/*R*luc ratio. n = 4. **(D) **BRET ratios were compared at identical total YFP/*R*luc ratio and corrected by subtracting the background BRET ratio calculated for unfused YFP and CA-p2-NC-p1-*R*luc co-expression. The corrected BRET ratio between CA-p2-NC-p1-*R*luc and Stau1^55^-YFP was arbitrarily set to 100%. n = 4. **(E) **293T cells were co-transfected with Pr55^Gag ^and wild-type or N-terminally truncated Stau1-Flag expressors. Twenty-four hours post-transfection, cell extracts were prepared and subjected to immunoprecipitation using anti-Flag antibodies. Cell lysates and immune complexes were analyzed by Western blotting using anti (α)-Flag and anti (α)-CA antibodies. Anti (α)-GAPDH antibodies were used as a loading control. n = 2.

To test the ability of Stau1^ΔNt88 ^to interact with Pr55^Gag^, we performed BRET assays between Stau1^ΔNt88^-YFP and a truncated Pr55^Gag ^(CA-p2-NC-p1-*R*luc) that was previously shown by the co-immunoprecipitaton assay to interact as efficiently with Stau1 as full-length Pr55^Gag ^[52] and (Figure 5A). To verify efficiency between these molecules, Stau1^ΔNt88^-YFP and Stau1^55^-YFP were expressed (Figure 5B) and the BRET saturation profiles determined (Figure 5C). Curves obtained with Stau1^55^-YFP and with Stau1^ΔNt88^-YFP were almost similar suggesting that Stau1^ΔNt88^mutant retains its capacity to bind to Pr55^Gag^. The BRET ratios were specific since the Gag-binding deficient mutant Stau1^ΔdsRBD3^-YFP showed reduced BRET ratios. In independent saturation experiments (Figure 5D), the specific BRET ratio following co-expression of Stau1^ΔNt88^-YFP and CA-p2-NC-p1-*R*luc was comparable [105.7 (+/- 18.1 S.D.)% of CA-p2-NC-p1/Stau1^55 ^corrected BRET ratio] to that obtained with wild type Stau1^55^-YFP at similar total YFP/*R*luc ratio. We could not detect a specific BRET signal when Stau1^F135A^-YFP [52] was used [1.3 (+/- 22.1 S.D.)% of CA-p2-NC-p1/Stau1^55 ^corrected BRET ratio].

The ability of Stau1^ΔNt88 ^to associate with Pr55^Gag ^was confirmed in co-immunoprecipitation assays (Figure 5E). Pr55^Gag ^and flag-tagged Stau1 or Stau1^ΔNt88 ^were co-expressed in 293T cells (Figure 5E, left panel) and proteins in the cell extracts were immunoprecipitated using anti-flag antibody. Western blot analyses of the immune complexes showed that Pr55^Gag ^successfully co-precipitated in a specific manner with both Stau1^55^-flag and Stau1^ΔNt88^-FLAG (Figure 5E, right panel). These results show that, although Stau1^ΔNt88 ^is unable to stimulate Pr55^Gag ^multimerization, its interaction with Pr55^Gag ^was maintained. This result suggests that Stau1 association to Pr55^Gag ^is not sufficient to influence HIV-1 assembly and that Stau1 N-terminus contains a regulatory element that is important for its function during this process.

### Stau1^ΔNt88^-HA_3 _does not enhance the assembly of membrane-associated Pr55^Gag ^complexes

**Figure 6:**
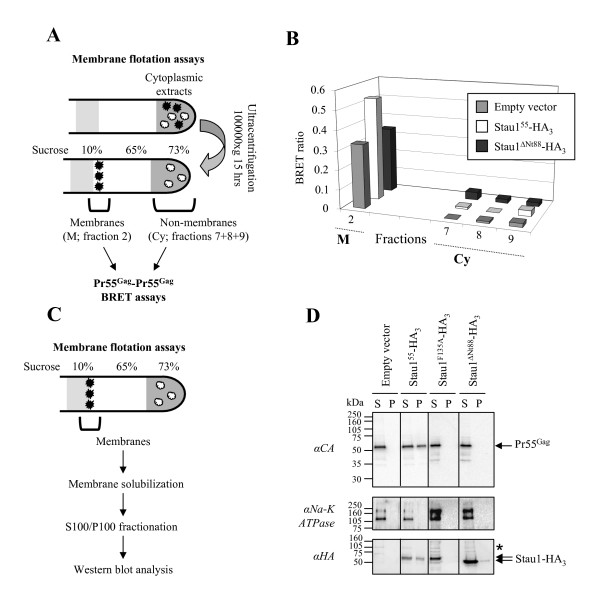
**Stau1^ΔNt88^-HA_3 _does not affect the multimerization of membrane-associated Pr55^Gag^**. **(A) **Schematic representation of the experimental procedure. 293T cells were transfected with Pr55^Gag^-*R*luc, Pr55^Gag^-YFP and Stau1-HA_3 _expressors. 48 hours post-transfection, cytoplasmic extracts were prepared and subjected to the membrane flotation assay. **(B) **Pr55^Gag^/Pr55^Gag ^BRET ratio was determined in each collected fraction. BRET ratios in fractions 1, 3, 4, 5 and 6 were omitted because *R*luc activity in these fractions was too low to provide a reliable BRET ratio. **(C) **Schematic representation of the experimental procedure. 293T cells were transfected with HxBRU PR-provirus and Stau1-HA_3 _expressors. 24 hours post-transfection, cytoplasmic extracts were prepared and subjected to the membrane flotation assay. Membrane fractions were collected and treated with 0.5% Triton X-100 during 5 minutes at room temperature in order to solubilize membranes. Resulting samples were subjected to ultracentrifugation at 100,000 × g during 1 hour. **(D) **Resulting supernatants (S100; S) and pellets (P100; P) were analyzed by Western blotting using anti (α)-CA, anti (α)-Na-K ATPase and anti (α)-HA antibodies. *: Non-specific labelling typically obtained with the anti-HA antibody.

We previously showed that the Stau1-mediated enhancement of Pr55^Gag ^multimerization occurs in membrane compartments [51]. Therefore, to test whether Stau1^ΔNt88^-HA_3 _reaches the membranes and whether the whole cell analysis described above masked an effect of Stau1^ΔNt88^-HA_3 _on assembly, membrane-associated virus assembly was analyzed in the context of Stau1^ΔNt88^-HA_3 _or Stau1^55^-HA_3 _over-expression. Cytoplasmic extracts from transfected 293T cells were analyzed by the membrane flotation assay (Figure 6A)[51]. This assay allows the separation of membrane-associated complexes (fraction 2; M) from the cytosolic ones (fractions 7, 8 and 9; Cy). First, Western blot analysis indicated that Stau1^ΔNt88^-HA_3 _was both over-expressed and present in membranes at the same levels as Stau1^55^-HA_3 _(data not shown). As previously described [51], Pr55^Gag^/Pr55^Gag ^BRET was readily detected in the membrane fraction (BRET ratio of 0.33) but not in the cytosolic fractions consistent with the fact that HIV-1 assembly occurs on cellular membranes (Figure 6B)[10,61,62]. Moreover, as reported before, Stau1^55^-HA_3 _over-expression led to an increase of 1.6-fold in the Pr55^Gag^/Pr55^Gag ^BRET ratio in the membrane fraction but not in the cytosolic fraction [51]. In contrast, there was little change in the ability of Pr55^Gag ^to multimerize in the membrane fraction when the mutant Stau1^ΔNt88^-HA_3 _was over-expressed.

The inability of Stau1^ΔNt88^-HA_3 _to modulate Pr55^Gag ^multimerization was then tested in the context of provirus-driven immature HIV-1 production. We had previously shown that Stau1-mediated increase in Pr55^Gag ^multimerization correlated with a partial resistance to mild detergent treatment of membrane-associated Pr55^Gag ^complexes [51]. Stau1 expressors and the protease-defective provirus HxBRU PR-were cotransfected in 293T cells. Forty hours post-transfection, membrane flotation assays on cytoplasmic extracts were performed and fractions containing membrane-associated complexes were collected. The resulting complexes were then subjected to ultracentrifugation at 100,000 × g for 1 hour (Figure 6C). In this assay, insoluble or high-density complexes are found in the pellet (P100) whereas proteins that are soluble or components of small complexes are retained in the supernatant (S100). Resulting P100 and S100 fractions were analyzed by Western blot. As previously shown [51], Pr55^Gag ^as well as the membrane marker, sodium potassium (Na-K) ATPase, were primarily found in the P100 fraction because Pr55^Gag ^was membrane-associated (data not shown). This was observed whether Stau1 proteins were over-expressed or not (data not shown). To separate Pr55^Gag ^complexes from membranes, membranes were solubilized with 0.5% Triton-X100. These experiments were done at room temperature to also solubilize lipid rafts and other membrane compartments that are detergent-resistant at 4°C. As reported before, membrane solubilization prior to S100/P100 fractionation resulted in a complete shift of Pr55^Gag^complexes and Na-K ATPase into the S100 fraction (Figure 6D)[51]. Stau1^55^-HA_3 _overexpression led to a partial resistance of 33% of Pr55^Gag ^complexes to Triton-X100 treatment, likely as a consequence of enhanced Pr55^Gag ^multimerization (Figure 6D). In contrast, Stau1^ΔNt88^-HA_3_, as well as Stau1^F135A^-HA_3 _used as control [51,52] failed to increase the density of Pr55^Gag ^complexes. Altogether, these results support the conclusion that the N-terminus of Stau1 is required for Stau1-mediated increase of Pr55^Gag ^assembly during HIV-1 assembly.

### Amino acids 26 to 37 of Stau1^55 ^are important for its function in Pr55^Gag ^multimerization

**Figure 7:**
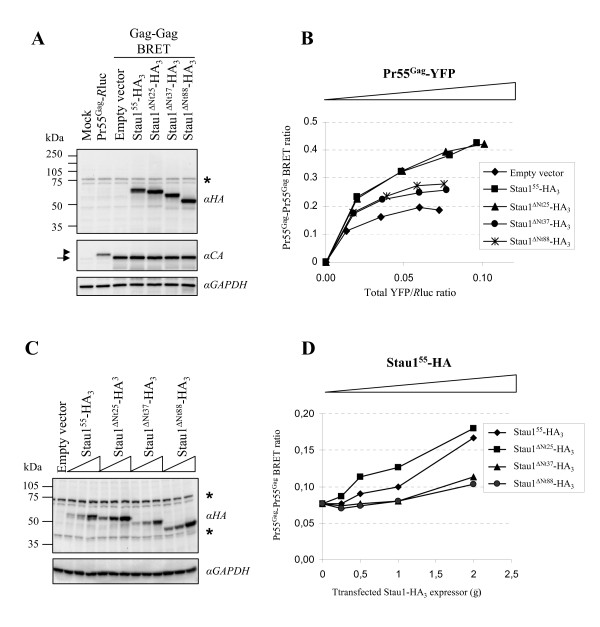
**Identification of the N-terminal region of Stau1^55 ^as a regulatory sequence for HIV-1 assembly**. **(A, B) **293T cells were transfected as described in Figure 4B but included additional Stau1^55^-HA deletion mutants. **(A) **For each condition, an aliquot of the cells (providing equivalent total YFP/*R*luc ratio) was used for Western blot analysis using anti (α)-HA and anti (α)-CA antibodies. Anti (α)-GAPDH antibody was used as a loading control. **(B) **Another aliquot of the cells was used for BRET assays. Calculated BRET ratios were plotted as a function of the corresponding total YFP/*R*luc ratio. **(C, D) **Dose response pr55^Gag^/pr55^Gag ^BRET assays. 293T cells were transfected with fixed amounts of pCMV-pr55^Gag^-*R*luc and pCMV-pr55^Gag^-YFP and increasing amounts of different Stau1-HA expressors. **(C) **48 hours later, half of the cells were lysed and analyzed by Western blotting using anti (α)-HA and anti (α)-GAPDH antibodies. *: Non-specific labelling.**(D) **The other half of the cells was used for BRET assays. BRET ratio is plotted as a function of the corresponding amount of transfected Stau1-HA expressor.

To more precisely map the functional determinant within the N-terminal region that is involved in the regulation of Pr55^Gag ^multimerization, we generated two additional deletion mutants, Stau1^ΔNt25^-HA_3 _and Stau1^ΔNt37^-HA_3 _and confirmed their expression (Figure 7A). Using the Pr55^Gag^/Pr55^Gag ^BRET assay in live 293T cells, Stau1^ΔNt25^-HA_3 _enhanced the BRET ratio like wild type Stau1^55^-HA_3 _suggesting that the first 25 amino acids are not required for this function (Figure 7B). In contrast, Stau1^ΔNt37^-HA_3 _did not significantly enhance the BRET ratio and generated a BRET curve similar to that obtained with Stau1^ΔNt88^-HA_3_. Expression of increasing amounts of Stau1^55^-HA_3 _or Stau1^ΔNt25^-HA_3 _(Figure 7C) led to a comparable increases of Pr55^Gag^/Pr55^Gag ^BRET ratio up to 2.16–2.34-fold (Figure 7D). In contrast, expression of Stau1^ΔNt37^-HA_3 _or Stau1^ΔNt88^-HA_3 _had no effect on Pr55^Gag^/Pr55^Gag ^BRET ratio when their expression is relatively low. However, when highly expressed, they slightly enhanced the BRET ratio by 1.34–1.47-fold. These results suggest that the sequence located between amino acids 26 and 37 is important for Stau1-mediated enhancement of Pr55^Gag ^multimerization and show that Stau1 acts on Pr55^Gag ^multimerization in a dose-dependent manner.

### Effect of Stau1 N-terminal mutants on VLP production

**Figure 8:**
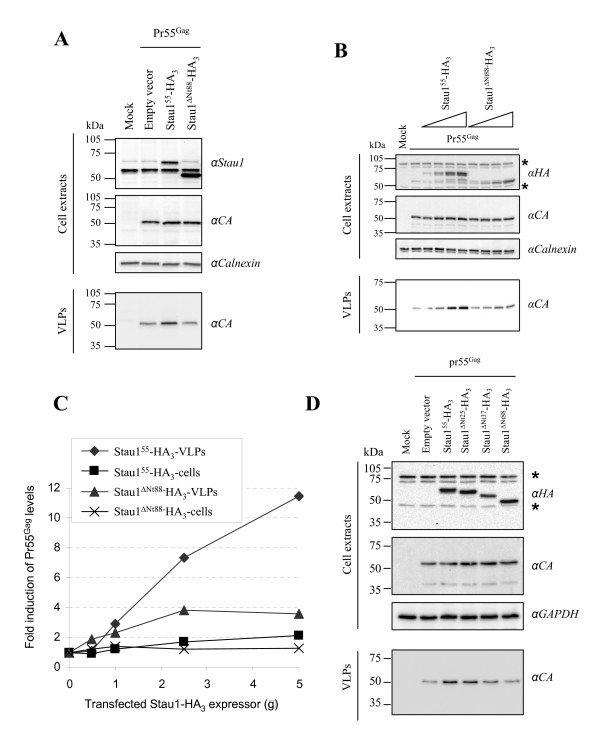
**Over-expression of Stau1-HA_3 _lacking amino acids 26 to 37 does not stimulate VLP production**. 293T cells were transfected with a Rev-independent Pr55^Gag ^expressor and constant **(A) **or increasing **(B) **amounts of either Stau1^55^-HA_3 _or Stau1^ΔNt88^-HA_3_-expressing plasmids. Twenty-four hours post-transfection, cells extracts and VLPs were prepared (see "Methods" section) and analyzed by Western blotting using anti (α)-Stau1, anti (α)-HA and anti (α)-CA antibodies. Anti (α)-calnexin antibodies were used as a loading control. *: Non-specific labelling. **(C) **Amounts of Pr55^Gag ^in cell extracts and in VLP were quantitated using Quantity One (version 4.5) software (Bio-Rad) and plotted as a function of the amounts of transfected Stau1^55^-HA_3 _and Stau1^ΔNt88^-HA_3_-expressors. (**D**) Cells were transfected as in (A) with two additional N-terminal Stau1 mutants. Cell extracts and VLPs were prepared and analyzed as in (A).

We already showed that Stau1 over-expression, likely as a consequence of its role in multimerization, also increases VLP release from the cell [51]. Therefore, considering the inability of Stau1^ΔNt88 ^to influence Pr55^Gag ^multimerization (Figure 4), we suspected that over-expression of Stau1^ΔNt88 ^would not stimulate VLP production. To test this idea, Pr55^Gag ^and either Stau1^55^-HA_3 _or Stau1^ΔNt88^-HA_3 _were co-expressed in 293T cells. Twenty-four hours post-transfection, the levels of VLP release were analyzed by Western blotting (Figure 8A). As expected, a 2-fold increase in the expression of Stau1^55^-HA_3 _as compared to endogenous Stau1 resulted in a 2.5-fold increase in VLP release while the cellular level of Pr55^Gag ^remained unchanged. In contrast, over-expression of Stau1^ΔNt88^-HA_3 _did not stimulate VLP production. To confirm these results, dose-response assays were performed. Constant amounts of Pr55^Gag ^were co-expressed with increasing amounts of either Stau1^55^-HA_3 _or Stau1^ΔNt88^-HA_3 _(Figure 8B). As previously shown [51], Stau1^55^-HA_3 _over-expression stimulated VLP production in a dose-dependent manner up to 10-fold with no significant change in the intracellular level of Pr55^Gag^. In contrast, Stau1^ΔNt88^-HA over-expression did not lead to a significant increase of VLP release, except at high expression levels (Figure 8C). However, the stimulation of VLP production by Stau1^ΔNt88^-HA_3 _was always less than that produced by Stau1^55^-HA_3_, when expressed at the same level. Analyses of VLP production in the presence of Stau1^ΔNt25^-HA and Stau1^ΔNt37^-HA indicated that, whereas Stau1^ΔNt25^-HA enhanced VLP release, Stau1^ΔNt37^-HA did not (Figure 8D) consistent with the conclusion that Stau1 molecular determinant for enhanced Pr55^Gag ^multimerization is carried by amino acids 26 to 37.

## Discussion

We previously reported that Stau1 participates in HIV-1 assembly by influencing Pr55^Gag ^multimerization [51]. However, very little is known about the molecular mechanisms underlying this process. In this report, we show that, in addition to Pr55^Gag^-binding via its dsRBD3 [51,52], Stau1's effect on Pr55^Gag ^multimerization depends on amino acids located within its N-terminus. Moreover, we show important contributions from both NC zinc fingers for the Stau1/Pr55^Gag ^interaction suggesting that Stau1 influences processes that depend on these NC sub-domains.

HIV-1 Gag mutants whose zinc fingers were disrupted, failed to interact with Stau1 as seen in BRET and co-immunoprecipitation assays (Figure 2). This suggests that Stau1 directly makes contact with these structures although we cannot rule out the possibility that these combined mutations affect the structure of other sub-domains of NC and potentially a Stau1-binding motif. Interestingly, viruses that harbour these mutations do not encapsidate Stau1 [53]. Although this phenotype could be attributed to the loss of HIV-1 genomic RNA packaging and to Stau1 RNA-binding activity, this strongly suggests that Stau1 encapsidation into HIV-1 may be achieved by the formation of a Stau1-Gag-RNA ternary complex where Stau1 interactions with both Pr55^Gag ^and genomic RNA contribute to this process [51,52].

NC zinc fingers are known to play important roles during several steps of the HIV-1 replication cycle such as genomic RNA packaging and reverse transcription [27-29,63]. In addition, they have been shown to be involved in Pr55^Gag ^assembly [5,6,29,30,46,64]. Our results with the BRET assay that monitors direct interaction between wild type and mutated Pr55^Gag ^proteins are consistent with these results. Whereas some studies reported dramatic defects in assembly and particle production only when both zinc fingers were disrupted [29,46], other showed that mutations in either of the NC zinc fingers (especially the C-terminal one) impaired HIV-1 assembly [30]. It is possible that the loss of one zinc finger can be compensated by the intact one and would explain why, in certain studies, single mutation within these motifs have no major effects on HIV-1 assembly. Interestingly, mutations in both zinc fingers are required for a complete loss of interaction between Stau1 and Pr55^Gag^, mutation in one zinc finger causing only a partial reduction. Therefore, through its interaction with the zinc fingers, Stau1 most likely influences steps of assembly that are controlled by the NC zinc fingers. In addition, we do not exclude the possibility that Stau1 also participates in other steps of HIV-1 life cycle that depend on NC [65].

Although both Stau1 and the Gag-NC zinc fingers are engaged in specific interactions with the genomic HIV-1 RNA, we do not have any evidence that Stau1 interacts with NC via the genomic RNA since the Stau1-Pr55^Gag ^association was shown to be resistant to RNase treatment [52]. Moreover, although the C15/49S NC mutant retains its ability to bind RNA nonspecifically through its basic amino acids [24-26], this mutant is unable to recruit Stau1 supporting the idea that Stau1/Gag-NC interaction is not bridged by RNA. Nevertheless, it is possible that Stau1-Gag-NC interactions favour recruitment of the genomic RNA and its subsequent trafficking and encapsidation. Indeed, mutating the conserved CCHC residues of the NC zinc fingers drastically impairs genomic RNA packaging in newly formed virions and that of Stau1 [53]. We previously showed that Stau1 association with HIV-1 genomic RNA is required for its subsequent encapsidation into the viral particle [53], our results now suggest that Stau1/Gag-NC interaction is also a critical determinant for this process. In addition, whether Stau1 interacts first with NC or with the genomic RNA to recruit the other partner or independently interacts with each of them through different pathways during HIV-1 assembly and RNA packaging processes is still unknown. The identification of a Stau1 mutant that retains its ability to associate with genomic RNA but is defective for Pr55^Gag ^binding will help answer these questions.

We identified the first 88 amino acids of Stau1 as a regulatory motif of its activity during HIV-1 assembly. Indeed, whereas Stau1^ΔNt88^-HA_3 _mutant is still able to interact with Pr55^Gag^, it fails to enhance Pr55^Gag ^multimerization as seen by BRET, fractionation and VLP release assays. These results strongly suggest that Stau1-binding to Pr55^Gag ^is not sufficient to influence HIV-1 assembly. They eliminate the possibility that the observed increase in the Pr55^Gag^/Pr55^Gag ^BRET ratio upon Stau1^55^-HA_3 _over-expression was the result of non-specific changes in the proximity of *R*luc and YFP tags due to Stau1 recruitment towards assembly complexes. Similarly, the possibility of major overall structural changes in Stau1^ΔNt88^-HA_3 _can be ruled out. Indeed, the Stau1^ΔNt88^-HA_3 _mutant retains its capacity to homo-dimerize, to enhance translation of repressed mRNAs, to bind ribosomes and to associate with membranes (unpublished data). Consequently, this sequence probably confers highly specific functions to Stau1 that are advantageous for HIV-1 assembly. It will be interesting to study Stau1^ΔNt88 ^encapsidation into HIV-1 as a means to determine whether the Stau1-mediated enhancement of Pr55^Gag ^multimerization is a prerequisite for its intraviral packaging or whether it relies on its association with HIV-1 genomic RNA and on the control of RNA selection for encapsidation [53].

How does this region control Stau1 activity? It is likely that Stau1, in conjunction with its Pr55^Gag^-binding activity, attracts host factors to Pr55^Gag ^complexes that are crucial for assembly. NC-associated proteins that are also important for the transition between specific Pr55^Gag ^assembly intermediates such as ABCE1 [44,45,47] are good candidates. Although ABCE1/Pr55^Gag ^interaction depends on NC basic amino acids [46], the disruption of both NC zinc fingers resulted in the loss of ABCE1/Pr55^Gag ^association by 80% [46]. Thus, it will be important to elucidate the functional relationship between Stau1 and NC-associated proteins to determine if their respective acquisition by assembly intermediates and functions during HIV-1 assembly are simultaneous or sequential.

Within the first 88 N-terminal amino acids of Stau1, a region of 12 amino acids (M^26^RGGAYPPRYFY^37^) controls Stau1 functions in regard to Pr55^Gag ^assembly. Post-translational modifications are very common among RNA-binding proteins such as hnRNPs and RNA helicase A and are known to regulate their cellular proteome, function and localization [66-69]. It is then conceivable that post-translational modification of Stau1's N-terminal region controls the recruitment of protein partners and/or modulates Stau1 function during HIV-1 assembly. Alternatively, the N-terminal sequence may recruit ubiquitin ligase through two potential ESCRT targeting domains (PPRY and YPFPVPPL) [50]. In most retroviruses (except HIV-1), the PPXY motif in Gag recruits a ubiquitin ligase and is required for virus budding and release [50,70,71]. Resulting ubiquitination allows targeting of the PPXY-containing protein to the ESCRT machinery located to the multivesicular bodies. Similarly, the YPX(n)L domain that is also present in some retroviruses (including HIV-1) recruits the AIP1/ALIX protein that also targets the cargo to the ESCRT [50,72,73]. Although Stau1^ΔNt88^-HA_3 _associates with membrane as efficiently as Stau1^55^-HA_3 _(data not shown), it is conceivable that these signals control the localization of Stau1 to specific membrane compartments to support HIV-1 assembly. Interestingly, Popov *et al. *recently showed that AIP1/ALIX, through its Bro1 domain, is able to bind the NC domain of Pr55^Gag ^in addition to p6 [64]. Strikingly, this interaction requires the integrity of both zinc fingers and is RNA-independent, as observed for Stau1/Pr55^Gag ^association. When over-expressed, AIP1/ALIX rescues the release defect of late domain-mutated HIV-1. Although AIP-1/ALIX over-expression has no effect on wild type HIV-1 release [74], in contrast to Stau1 [51], it is possible that Stau1 and AIP-1/ALIX, through their simultaneous or sequential association with the NC zinc fingers, cooperate during HIV-1 assembly. The putative interplay between AIP1/ALIX, ESCRTs and Stau1 during both wild type and L-domain-mutated virus assembly will be very interesting to explore in future studies.

Finally, our work highlights the modular nature of the Stau1 protein in the following ways. The double-stranded RNA binding domain (dsRBD3) interacts with Pr55^Gag ^[52] whereas the N-terminus controls its activity during HIV-1 assembly. This is supported by the fact that Stau1^Δ88^-HA_3 _still associates with HIV-1 Gag (Figure 5) but fails to enhance its multimerization (Figures 4, 6, 8). Strikingly, Drosophila Stau1 orthologue, dStau, has also been shown to function as a modular protein [75]. The third dsRBD of dStau for example is involved in dStau association to *oskar *RNA whereas the second dsRBD is important for the microtubule-dependent transport of the mRNP and the fifth dsRBD is involved in the derepression of *oscar *mRNA translation, once localized [75].

## Conclusion

In this study, we provide important new information about the determinants in both Stau1 and Pr55^Gag ^that impact on HIV-1 assembly.

## Authors' contributions

LCC participated in the design of the study, carried out most of the experiments and drafted the manuscript.

KB carried out the immunoprecipitation experiments shown in Figures 2D and 5E, contributed to figure 3B and 8D, reproduced the experiment shown in figure 8A and generated the Flag constructs.

AJM participated in the design and coordination of the study and critically revised the manuscript.

LDG participated in the design and coordination of the study and wrote the final version of the manuscript.

All authors read and approved the final manuscript.
